# FMO3 deficiency of duck leads to decreased lipid deposition and increased antibacterial activity

**DOI:** 10.1186/s40104-022-00777-1

**Published:** 2022-11-16

**Authors:** Xingzheng Li, Jianlou Song, Xuefeng Shi, Mingyi Huang, Lei Liu, Guoqiang Yi, Ning Yang, Guiyun Xu, Jiangxia Zheng

**Affiliations:** 1 College of Animal Science and Technology, China Agricultural University, Beijing, 100193 China; 2 Shenzhen Branch, Guangdong Laboratory of Lingnan Modern Agriculture, Genome Analysis Laboratory of the Ministry of Agriculture and Rural Affairs, Agricultural Genomics Institute at Shenzhen, Chinese Academy of Agricultural Sciences, Shenzhen, 518124 China

**Keywords:** Antibacterial, Cardiovascular disease (CVD), Duck, Flavin-containing monooxygenase 3 (FMO3), Metagenome, Proteome, Transcriptome, Trimethylamine (TMA)

## Abstract

**Background:**

Most duck eggs possess a fishy odor, indicating that ducks generally exhibit impaired trimethylamine (TMA) metabolism. TMA accumulation is responsible for this unpleasant odor, and TMA metabolism plays an essential role in trimethylaminuria (TMAU), also known as fish odor syndrome. In this study, we focused on the unusual TMA metabolism mechanism in ducks, and further explored the unclear reasons leading to the debilitating TMA metabolism.

**Methods:**

To achieve this, transcriptome, proteome, and metagenome analyses were first integrated based on the constructed duck populations with high and low TMA metabolism abilities. Additionally, further experiments were conducted to validate the hypothesis regarding the limited flavin-containing monooxygenase 3 (FMO3) metabolism ability of ducks.

**Results:**

The study demonstrated that liver FMO3 and cecal microbes, including *Akkermansia* and *Mucispirillum*, participated in TMA metabolism in ducks. The limited oxidation ability of FMO3 explains the weakening of TMA metabolism in ducks. Nevertheless, it decreases lipid deposition and increases antibacterial activity, contributing to its survival and reproduction during the evolutionary adaptation process.

**Conclusions:**

This study demonstrated the function of FMO3 and intestinal microbes in regulating TMA metabolism and illustrated the biological significance of FMO3 impairment in ducks.

**Supplementary Information:**

The online version contains supplementary material available at 10.1186/s40104-022-00777-1.

## Introduction

Since the 1970s, trimethylamine (TMA) metabolism disorders have been observed in numerous species, including humans [1]. TMA metabolism disorder in humans, commonly known as trimethylaminuria (TMAU) or fish odor syndrome, is mainly characterized by abnormally high TMA levels in urine, sweat, and expired air [2]. This defect causes a TMA-derived unpleasant fishy smell, leading to psychosocial issues such as depression, social isolation, and even suicide [3–6]. Exon mutations in flavin-containing monooxygenase 3 (*FMO3*) primarily cause TMAU. TMA is preliminarily derived from intestinal degradation and oxidized to trimethylamine N-oxide (TMAO) by the liver FMO3 enzyme. However, FMO3 deficiency leads to decreased or even reduced protease activity, resulting in the inability to oxidize TMA [7, 8]. Thus, excessive fishy-smelling TMA is produced and excreted by affected individuals. Although some treatments might help relieve the symptoms, there is currently no cure for TMA metabolism disorders [9]. Revealing the mechanism of TMA metabolism could promote a deeper understanding of these issues and provide novel perspectives on TMAU treatment.

TMA metabolism disorders are considered abnormal symptoms in humans, livestock, and poultry [10–12]. Additionally, studies have discovered that these disorders result from *FMO3* exon mutations in animals other than humans, such as cows, chickens, and quails [13–15]. However, an exception is that ducks generally experience TMA metabolism disorders as “normal symptoms”. A study reported that the fishy odor intensity of duck eggs was stronger, and the TMA content was higher than that of its genetically close species, chicken [16]. Although ducks show different TMA metabolic patterns, their mechanisms and causes remain unclear. Investigating the TMA metabolism process in ducks may provide a deeper understanding of the origin and pathogenesis of TMA metabolism disorders. Moreover, since high body fat does not lead to any health problems, the close relationship between cardiovascular disease (CVD) and the TMA oxidation product TMAO makes it interesting to investigate its TMA metabolism process [17–21].

To investigate the TMA metabolism process in ducks, provide further insight into the treatment for patients with TMAU, and explore the effect of diminished function of FMO3 on evolutionary adaption. We constructed a duck population with high TMA and low TMA metabolism abilities. Transcriptome, proteome, and metagenome analyses were then integrated to provide a comprehensive view of the mechanism of TMA metabolism in ducks. In addition, the different effects of FMO3 between ducks and chickens were compared. The effects of FMO3 deficiency on lipid deposition and antibacterial activity were also evaluated. Further studies are needed to validate our hypothesis.

## Materials and methods

### Animals and experimental design

To construct a duck population with high and low TMA metabolism abilities, 294 ducks (Jingjiang, 72 weeks) were fed a diet containing 4000 mg/kg choline chloride (TMA precursor), and eggs were collected until the sixth day after choline supplementation. Then, tissues including the liver and ceca of these 294 ducks were collected, and samples were immediately snap-frozen in liquid nitrogen and stored at − 80 °C.

TMA in egg yolks and cecal contents was quantified using headspace gas chromatography (HS-GC). For transcriptomic analysis, six liver samples with low egg TMA levels, six liver samples with high egg TMA levels, and 12 random liver samples were selected for RNA-Seq sequencing. For proteomic analysis, four liver samples with low egg TMA levels and four liver samples with high egg TMA levels were selected for tandem mass tags (TMT) liquid chromatography-mass spectrometry (LC-MS/MS). Finally, for metagenomic analysis, 30 cecal content samples with low cecal TMA levels and 30 cecal content samples with high cecal TMA levels were selected for 16S rRNA V4 sequencing.

For FMO3 enzyme comparison between ducks and chickens, livers of duck (Jingjiang) and chicken (White Leghorn) were used for FMO3 immunohistochemical (IHC) staining. In addition, ducks (Jingjiang), chickens (AA, AT, and TT of CAU-3), and chickens (White Leghorn) were used for FMO3 enzyme activity measurements. CAU-3 chickens were genotyped for an A/T polymorphism at position 1034 of the chicken *FMO3* exon (c.984 A > T, accession number: AJ431390), and the TT genotyped individuals had impaired FMO3 function.

For the verification experiment, lipid deposition was evaluated between duck and chicken species at both young and old age. Ducks (Jingjiang) 17 and 70 weeks old, chickens (White Leghorn) 18 weeks old, and chickens (AA, AT, and TT of CAU-3) 62 weeks old were selected, and aortas and livers were used for Oil Red O and hematoxylin staining. Additionally, an antibacterial test for TMA was performed using *Escherichia coli* (*E. coli*).

In the present study, samples with low TMA levels were represented by CL, and TH represented samples with high TMA levels.

### TMA quantification

TMA content was quantified using HS-GC as described previously with minor modifications [16, 22, 23]. In brief, 10 g (1 g cecal contents) yolk samples were weighed, and 10% trichloroacetic acid (TCA) solution was added to yield a total volume of 30 mL (10 mL). The sample tubes were vortexed thoroughly and allowed to stand overnight. The supernatant was filtered through Whatman #2 filter paper, and 1 mL of the filtrate was transferred into a headspace vial. Next, distilled water (5 mL) was added to the headspace vial, followed by the addition of 2 mL 50% KOH solution. The headspace vial was immediately sealed with a headspace cap, and the solution was vortexed thoroughly. Next, the headspace gas was injected into a 30 m × 0.32 mm × 0.25 μm Agilent CP8763 wax GC column. Detection was performed on an Agilent 7694E GC system (Agilent Technologies, Senta  Clara, CA, USA) equipped with a 7694E headspace sampler and a flame ionization detector. The TMA concentration was quantified using the standard curve method.

### RNA isolation and library preparation for RNA-Seq

The total RNA (six samples with low egg TMA levels, six samples with high egg TMA levels, and 12 random samples) was isolated from the right liver tissue per sample using Dynabeads™ mRNA Purification Kit (Thermo Fisher Scientific, Waltham, MA, USA). RNA concentration was determined using the Qubit®RNA Assay Kit in Qubit®2.0 (Thermo Fisher Scientific, Waltham, MA, USA). RNA integrity was assessed using the RNA Nano 6000 Assay Kit on the Bioanalyzer 2100 system (Agilent Technologies, Santa Clara, CA, USA).

Sequencing libraries were generated using the NEBNext® Ultra™ RNA Library Prep Kit for Illumina® (New England Biolabs, Ipswish, MA, USA) following the manufacturer’s recommendations. The libraries were sequenced on an Illumina HiSeq2000 platform generating 2 × 150 bp paired-end reads.

### RNA-Seq bioinformatic analyses

Raw reads were preprocessed using fastp for quality control [24]. Reads that passed quality control were subjected to further bioinformatics analyses. The processed reads were mapped to the duck (*Anas platyrhynchos*) reference genome assembly CAU_duck1.0 using HISAT2 with Ensembl genome annotation v98 [25]. Subsequently, the aligned reads were assembled and transcript abundances were quantified using StringTie in a guided approach, using the Ensembl genome annotation v98 and the duck (*Anas platyrhynchos*) reference genome assembly CAU_duck1.0 [26]. Differentially expressed transcripts were assessed (6 samples vs. 6 samples) using the Wald test implemented in the DESeq2 R package [27]. The apeglm method was used for effect size shrinkage, and the independent hypothesis weighting (IHW) was used for *P*-value adjustment of the DESeq2 results [28, 29]. Transcripts with an adjusted *P*-value < 0.05 were assigned as differentially expressed.

The weighted correlation network of the expression profile was built using the WGCNA R package with 24 samples [30]. First, the low-count transcripts were filtered out by keeping only transcripts with at least 1200 counts in all samples. Next, expression data were transformed using the variance stabilizing transformation (VST) method [31]. Then, the soft thresholding power, by which co-expression similarity is raised to calculate adjacency, was chosen based on the criterion an of approximate scale-free topology (R^2^ > 0.8). Adjacency was transformed into a topological overlap matrix (TOM), and the corresponding dissimilarity was calculated to minimize the effects of noise and spurious associations. A hierarchical clustering tree (dendrogram) of transcripts was produced using hierarchical clustering with branches corresponding to the transcript co-expression modules. Modules of co-expressed transcripts were identified using the dynamic tree-cut algorithm (cutHeight = 0.3). The correlation between transcripts and phenotypes was quantified as the gene significance (GS). For each module, the correlation of the module eigengene and transcript expression profile was measured as module membership (MM).

Genomic exon variations were detected using the GATK Best Practices workflow for calling variants in RNA-Seq (created on March 6, 2014, last updated on January 21, 2017), which consisted of the following steps: (i) mapping to the reference using the STAR 2-pass method; (ii) adding read groups, sorting, marking duplicates, and creating the index using the Picard tools; (iii) Split‘N’Trim and reassign mapping qualities using the SplitNCigarReads GATK tool; (iv) indel realignment and base recalibration; (v) variant calling with HaplotypeCaller; and (vi) variant filtering with VariantFiltration [32]. In addition, association analysis between exon variants and phenotypes was performed using PLINK v1.90 [33].

### Protein extraction, TMT labeling, and LC-MS/MS analysis

The tissues (four liver samples with low egg TMA levels and four liver samples with high egg TMA levels) were ground in liquid nitrogen, lysed using protein extraction buffer (8 mol/L urea, 0.1% SDS) containing 1 mmol/L phenylmethylsulfonyl fluoride (Beyotime Biotechnology, Shanghai, China) and protease inhibitor cocktail (Roche, Basel, Switzerland) on ice for 30 min, and then centrifuged at 16,000 × *g* for 15 min at 4 °C. The supernatant was collected, and the protein concentration was measured using a BCA assay (Pierce Biotechnology, MA, USA). The tissue lysates were stored at − 80 °C before further processing.

Protein samples were reduced in tris (2-carboxyethyl) phosphine (TCEP), alkylated in iodoacetamide (IAA), and digested using trypsin. TMT10plex™ (Pierce Biotechnology, MA, USA) was used as an isobaric tag for relative quantification, and TMT labeling was performed according to the manufacturer’s protocol. Briefly, 100 μg of each condition was transferred into a new tube, and 100 mmol/L triethylammonium bicarbonate (TEAB) buffer was added to the protein solution to a final volume of 100 μL. Then, 5 μL of 200 mmol/L TCEP was added, and the sample was incubated at 55 °C for 1 h, followed by the addition of 5 μL of 375 mmol/L IAA to the sample and incubation for 30 min in the dark at room temperature. Proteins were precipitated using pre-chilled (− 20 °C) acetone. After resuspension with 100 μL of 100 mmol/L TEAB, the proteins were digested overnight at 37 °C with 2.5 μg trypsin (Sigma-Aldrich, St. Louis, MO, USA). The digested samples were individually labeled with TMT10 reagents at room temperature for 1 h as follows: CL006, CL014, CL015, and CL030 were labeled with TMT10plex-127C, TMT10plex-127 N, TMT10plex-128C, TMT10plex-128 N, and TH292, TH293, TH294, and TH296 with TMT10plex-129C, TMT10plex-129 N, TMT10plex-130 N, and TMT10plex-131 N, respectively. The labeling reaction was quenched by the addition of 8 μL of 5% hydroxylamine. Finally, eight labeled peptide aliquots were combined for subsequent fractionation.

For fractionation of the labeled peptides, samples were first lyophilized and reconstituted in solvent A (2% ACN, pH 10). Then, the samples were loaded onto an Xbridge PST C18 Column, 130 Å, 5 μm, 250 mm × 4.6 mm column (Waters, Millford, MA, USA) and resolved by the basic reversed-phase liquid chromatography (RPLC) method using a gradient of 5% to 95% solvent B (90% ACN, pH 10) in 40 min. Forty fractions were collected, which were then concatenated to 20 fractions, vacuum-dried, and stored at − 80 °C before further LC-MS/MS analysis.

LC-MS/MS analysis was performed using a Q Exactive mass spectrometer (Thermo Fisher Scientific, Waltham, MA, USA). The peptide mixture was separated by reversed-phase chromatography on a DIONEX nano-UPLC system equipped with an Acclaim C18 PepMap100 nano-trap column (75 μm × 2 cm, 2 μm particle size) (Thermo Fisher Scientific, Waltham, MA, USA) connected to an Acclaim PepMap RSLC C18 analytical column (75 μm × 25 cm, 2 μm particle size) (Thermo Fisher Scientific, Waltham, MA, USA). The sample was dissolved in a sample buffer containing 4% acetonitrile and 0.1% formic acid. A linear gradient of mobile phase B (0.1% formic acid in 99.9% acetonitrile) from 3% to 30% in 43 min, followed by a steep increase to 80% mobile phase B in 1 min, was used at a flow rate of 300 nL/min. The nano-LC was coupled online with the Q Exactive mass spectrometer using a stainless steel emitter coupled to a nanospray ion source. Mass spectrometry analysis was performed in a data-dependent manner, with full scans (350–1600 *m/z* range) acquired using an Orbitrap mass analyzer at a mass resolution of 70,000 at 400 *m/z* in Q Exactive. The 20 most intense precursor ions from the survey scan were selected for MS/MS from each duty cycle and detected at a mass resolution of 35,000 at an *m/z* of 400 in the Orbitrap analyzer. All tandem mass spectra were obtained using the higher-energy collision dissociation (HCD) method. Dynamic exclusion was set to exclude previously sequenced precursor ions for 18 s.

### Proteomics bioinformatics analyses

LC-MS/MS mass spectrometry data (four samples vs. four samples) were searched against the Uniprot duck (*Anas platyrhynchos*) database (Proteome ID: UP000016666) using MaxQuant with an integrated Andromeda search engine [34, 35]. MaxQuant searches for global proteome analysis were carried out with the following parameters: trypsin as the proteolytic enzyme, with up to two missed cleavages for enzymatic cleavage, oxidation (M) and acetyl (protein N-term) as variable modifications, and carbamidomethyl as fixed modification. In addition, peptides were identified by applying 1% FDR at both peptide-spectrum matches (PSM) and protein levels.

Downstream MaxQuant analysis was performed using the Proteus R package [36]. Peptides were assigned to proteins based on the leading razor protein, and protein abundance was quantified using the high-flyers method [37]. The TMT proteomic data were normalized using the constrained standardization (CONSTANd) method [38]. Differential abundance analysis for the normalized proteomic data was performed using the limma R package; proteins with a *P*-value < 0.01 were differentially expressed [39]. Gene Ontology (GO) overrepresentation enrichment of differentially expressed proteins was performed using WebGestalt [40]. Finally, protein-protein interaction (PPI) networks were constructed using the STRING database [41]. The peptides analysis process is detailed in Supplementary Material (Fig. S1A-C).

### DNA extraction and sequencing for microbiome data

The total DNA (30 cecal content samples with low cecal TMA levels and 30 cecal content samples with high cecal TMA levels) was extracted using an OMEGA E.Z.N.A.® Stool DNA Kit (Omega Bio-Tek Inc., Norcross, GA, USA) according to the manufacturer’s instructions. The V4 region of 16S rRNA was amplified using 515F-806R primers with barcodes. The PCR was carried out using Phusion® High-Fidelity PCR Master Mix (New England Biolabs, Ipswich, MA, USA). Sequencing libraries were generated using TruSeq® DNA PCR-Free Sample Preparation Kit (Illumina, San Diego, CA, USA) following the manufacturer’s recommendations. Library quality was assessed on a Qubit® 2.0 Fluorometer (Thermo Fisher Scientific, Waltham, MA, USA) and Agilent Bioanalyzer 2100 system (Agilent Technologies, Santa Clara, CA, USA). The library was sequenced on an Illumina HiSeq2500 platform to generate 250 bp paired-end reads.

### 16S rRNA bioinformatic analyses

The 16S amplicon data (30 samples vs. 30 samples) were analyzed using QIIME2 software [42]. DADA2 was used to perform quality control, correct errors in marginal sequences, and remove chimeric sequences [43]. Alpha diversity was calculated using the observed OTUs. Beta diversity patterns were explored by performing the principal coordinates analysis (PCoA) with phylogeny-based (UniFrac) weighted distances between samples. The Silva_132_release database was used to assign taxonomy information based on a pre-trained naive Bayes classifier. Differences in abundance were tested using LEfSe and Mann-Whitney U tests [44]. All statistical analyses were performed with α = 0.05.

For co-occurrence network analysis, all possible Spearman’s rank correlations between OTUs present in more than 1/3 of the sequenced samples were calculated. Correlations with Spearman’s correlation coefficient (*ρ*) were > 0.4 and statistically significance (*P* < 0.05) was presented. Co-occurrence networks were explored and visualized using the interactive platform Gephi [45].

Enterotype analysis was performed using genus abundance, as described previously [46]. In brief, samples were clustered based on relative abundances using the Jensen-Shannon divergence (JSD) distance and partitioning around medoids (PAM) clustering algorithm. The optimal number of clusters was assessed using the Calinski-Harabasz (CH) index [47]. Finally, PCoA was performed to visualize distances.

### Multi-omics data integration

Procrustes analysis was performed using the vegan R package. First, the Monte Carlo *P*-values for rotational agreement significance testing were determined from 999 permutations. In addition, the cumulative intensities of both the protein quantity (by TMT) and mRNA were calculated. Finally, differentially expressed proteins (DEPs) and transcripts (DETs) were compared.

### Immunohistochemistry

The tissues were dehydrated in ascending concentrations of ethanol and xylene and finally embedded in paraffin. Tissue sections (10 μm) were cut on a rotary microtome, and mounted on gelatin subbed slides. The operational approach of immunohistochemistry was performed according to the previous method as Novick et al. described [48]. Antibodies against FMO3 for chickens and ducks were purchased from Living Biotechnologies Co., Ltd. (Living Biotechnologies, Beijing, China). The target sections were observed by light microscopy at × 40 magnification. A Canon EOS 7D digital camera (Canon, Tokyo, Japan) connected to the microscope was used to capture images.

### Determination of FMO3 enzyme activity

Liver microsomes were prepared by differential centrifugation of homogenates as described previously [49]. The protein content of liver microsomes was determined by a previously described method using crystalline bovine serum albumin as the standard [50]. FMO3 activity was determined using TMA, the N-oxygenation of typical FMO3 substrates, with minor modifications [51, 52]. A final volume of assay mixture (1 mL) contained 50 mmol/L Tris-HCl buffer (pH 7.4), an NADPH generating system (10 mmol/L MgCl_2_, 10 mmol/L glucose 6-phosphate, 0.4 mmol/L NADP^+^, 0.5 U of glucose-6-phosphate dehydrogenase), 500 μg of liver microsomal protein, and 3 mmol/L TMA. The TMAO production rates were determined indirectly after reduction with Fe-EDTA to free TMA. The TMA content of the mixture was analyzed using HS-GC. All the chemicals were obtained from Sigma-Aldrich (Sigma-Aldrich, St. Louis, MO, USA). The generated TMAO content per hour represented FMO3 enzyme activity.

### Aortic lesion assessment

The aortic lesion was quantified by en face analysis of the aorta (including the aortic arch, thoracic, and abdominal regions) and cross-sectional analysis of the lesion areas as previously described with modifications [53]. The aortic tissue samples were opened longitudinally and stained with Oil Red O (Wuhan Service Biotechnology, Hubei, China), and the lesion areas were quantified. En face images of the aorta were taken with a Canon EOS 7D digital camera (Canon, Tokyo, Japan) and analyzed using ImageJ software. To measure atherosclerotic lesions at the cross-sectional area of the aortic arch, a small segment of the aortic arch (in the same area) was embedded in optimal cutting temperature (OCT) compound (Sigma-Aldrich, St. Louis, MO, USA) and frozen at − 20 °C. Sections (8-μm thickness) were collected. Lesions in the cross-sectional area of the aortic arch were stained with Oil Red O and hematoxylin. For each section of the aortic arch, randomly located areas were assessed by light microscopy at × 20 magnification. A Canon EOS 7D digital camera (Canon, Tokyo, Japan) connected to the microscope was used to capture images. The stained area was quantified based on the ratio of the positive area to the atherosclerotic lesion.

### Liver histological observation

Sections were examined for steatosis using Oil Red O staining as previously described with modifications [54]. For cryo-section cutting, fixed samples were embedded in OCT (Sigma-Aldrich, St. Louis, MO, USA) in frozen, and then sectioned at 10 μm; all operations were carried out under frozen conditions. After that, samples were stained with Oil Red O (Wuhan service Biotechnology, Hubei, China), differentiated with isopropanol, washed with distilled water, and stained with hematoxylin. For each section of the liver, 10 randomly located areas were assessed using light microscopy at × 80 magnification. A Canon EOS 7D digital camera (Canon, Tokyo, Japan) connected to the microscope was used to take pictures. Then, the lipid deposition was quantified using ImageJ software.

### Determination of TMA antibacterial activity

First, 0, 40, and 80 μg/mL TMA agarose liquid medium (10 g tryptone, 5 g yeast extract, 10 g sodium chloride, and TMA hydrochloride) was prepared. Next, 200 mL of each TMA agarose liquid medium was transferred into conical flasks, and 100 μL of the 10^5^ CFU/mL *E. coli* solution was added to each conical flask. Then, conical flasks containing different concentrations of TMA were placed in a 37 °C incubator, and the optical density (OD) values of the bacterial solution were recorded per hour using a UV-vis spectrophotometer (AuCy Scientific Instrument, Shanghai, China) at OD = 600 nm.

The antibacterial activity of TMA was determined using a Two-Petri-dish assay [55]. The *E. coli* solution was diluted to 10, 10^2^ and 10^3^ CFU/mL with an agar nutrient liquid medium. The diluted *E. coli* solution was evenly coated on an agarose solid medium (10 g tryptone, 5 g yeast extract, 10 g sodium chloride, and 15 g agar/L). TMA solutions with the concentration of 1, 2, 3, 4, and 5 μg/mL were prepared with TMA hydrochloride (Macklin, Shanghai, China). Sterilized filter paper was placed into the cover of the Petri dish, and different concentrations of TMA solution were dripped. The dishes were immediately closed and sealed with parafilm. The Petri dishes were incubated upside down overnight at 37 °C, and then the colonies were counted and recorded.

## Results

### Liver FMO3 transcripts played a limited role in the TMA metabolism process of ducks

**Fig. 1:**
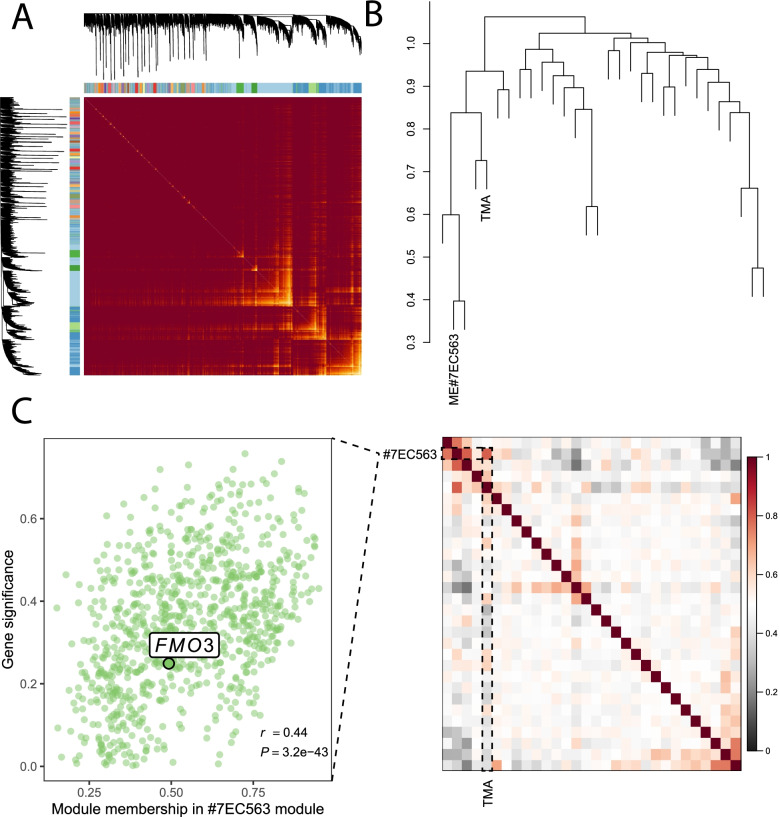
*FMO3* transcript participated in the TMA metabolism process with limited functions. **A** Heatmap of the transcripts network. The heatmap depicts the topological overlap matrix (TOM) among all transcripts. The light color represents high overlap and the dark red color represents low overlap. The transcript dendrogram and module assignment are shown along the left and top sides. **B** The eigengene network represents the relationships among modules and TMA production. The top panel shows a hierarchical clustering dendrogram of the eigengenes, and the bottom panel shows the eigengene adjacency. **C** The scatterplot of gene significance (GS) for TMA vs. Module Membership (MM) in the most significantly correlated module #7EC563

RNA-seq was used to investigate transcripts involved in TMA metabolism in ducks. For the weighted correlation network analysis (WGCNA), adjacency was calculated and transformed into a TOM using a soft threshold power β of 6 (Fig. S2A). Twenty-nine modules were identified from the filtered transcripts (Fig. 1A and B, Fig. S2B). Module #7EC563 was significantly associated with TMA deposition in duck eggs (Fig. 1C). Although genes in this module were not significantly enriched in the molecular function annotations, candidate *FMO3* transcripts were found in this module (Fig. 1C and Fig. S2C). In addition, differentially expressed transcripts were not enriched in any molecular function annotation (Fig. S3A-C). The transcripts of *FMO3* between the CL (samples with low TMA levels) and TH (samples with high TMA levels) groups were not significantly different, which was verified by qPCR (Table S1). Additionally, the association analysis results revealed that variants did not cause heavy TMA deposition in duck eggs within the *FMO3* gene regions (Fig. S4).

### Liver FMO3 protease regulates the TMA metabolism of ducks

**Fig. 2:**
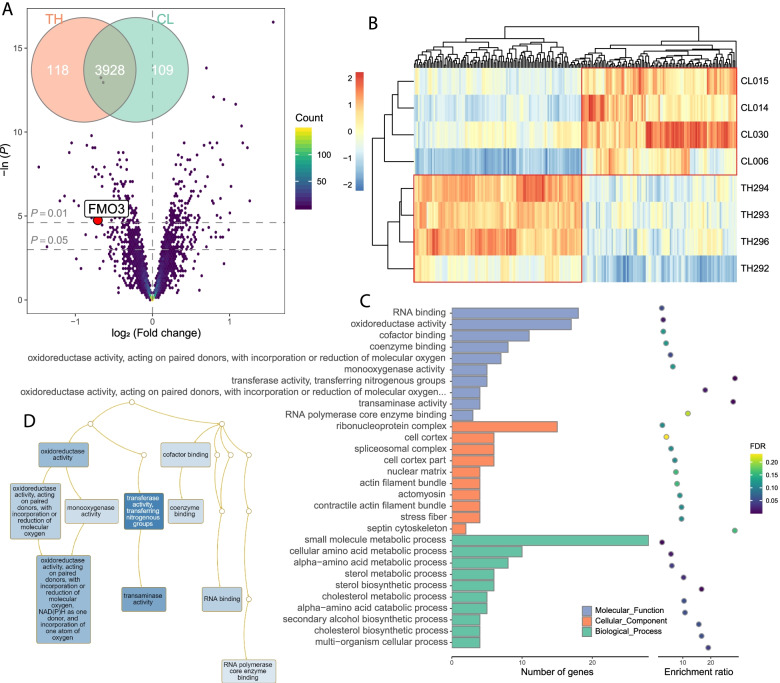
FMO3 protease in duck liver regulates TMA metabolism. **A** Volcano plot displaying the –ln (*P*-value) vs. log_2_ (TH/CL) for all quantified proteins with a Venn diagram displaying the differently expressed proteins (DEPs). **B** Heatmap of hierarchical clustering based on the DEPs. **C** Gene ontology enrichment categories for DEPs. **D** Directed acyclic graph (DAG) of DEPs in enriched molecular function category, deeper color represents higher significance

We identified 23,323 unique peptide fragments and quantified 4155 proteins. There were 227 differentially expressed proteins (DEPs), of which 109 were upregulated in the CL group, and 118 were upregulated in the TH group (Fig. 2A and B, Table S2). Molecular function annotation in GO functional analysis showed that 17 DEPs under low and high TMA conditions were mainly enriched in oxidoreductase activity and subterms (GO:0016491, GO:0016705, GO:0016709, and GO:0004497), suggesting that these proteins have oxidoreductase characteristics (Fig. 2C and D, Table S3). Particularly, the FMO3 protein expression level in the CL group was slightly higher than that in the TH group (Fig. 2A). We also searched the STRING database and obtained a protein-protein interaction (PPI) network of the DEPs, which might affect TMA metabolism (Fig. S1D).

### Akkermansia and Mucispirillum down-regulates and up-regulates TMA level, respectively

**Fig. 3:**
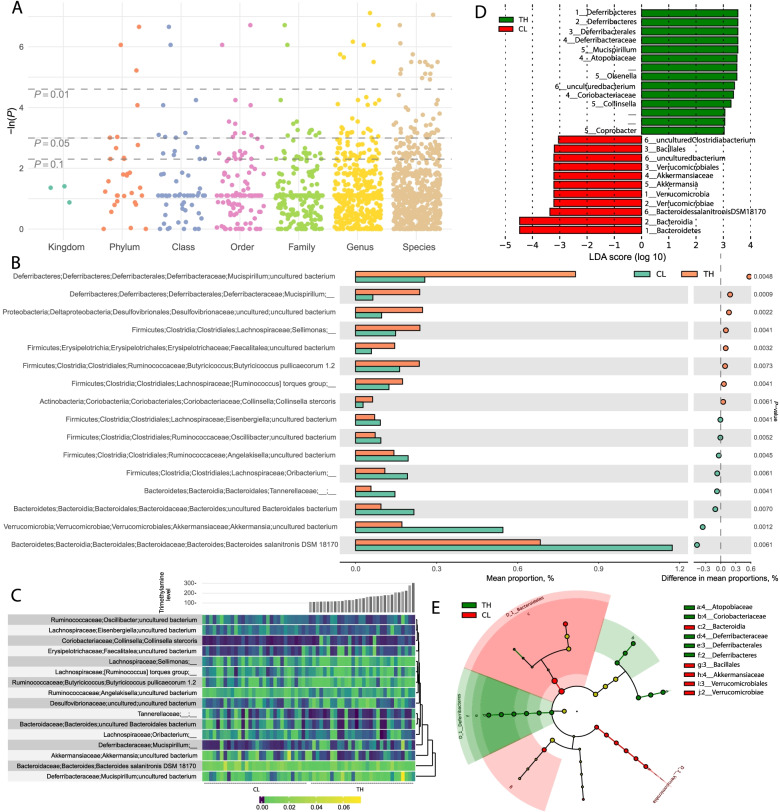
Microbiota in duck cecum regulates TMA metabolism. **A** Statistical significance of differences in bacterial abundance between the CL and TH ducks at the kingdom, phylum, class, order, family, genus, and species levels (Mann-Whitney U test, *P* < 0.01). **B** Characteristics of significantly different bacteria in abundance using Mann-Whitney U test. The left panel represents the relative abundance, and the right panel represents the difference and significance. **C** Details of significantly different bacteria using Mann-Whitney U test. The top panel represents the TMA distribution of cecal content, and the heatmap represents candidate bacterial abundance in each sample. **D** Differentially abundant bacteria were determined using the Kruskal-Wallis test (LEfSe analysis, LDA score > 3, *P* < 0.05). **E** Taxonomic cladogram of differentially abundant microbes using LEfSe analysis. The size of each node represents their relative abundance

Transcriptome and proteome results showed that liver FMO3 does not play a vital role in TMA regulation. We investigated the cecal microbiota structure associated with TMA metabolism in ducks using 16S rRNA V4 sequencing data, as TMA was mainly generated in the cecum. Overall, the bacterial richness, distribution, and diversity of the cecal microbiota exhibited similar patterns in CL and TH ducks (Fig. S5A-C). To further identify the specific bacterial taxa associated with TMA metabolism, we compared the composition of the cecal microbiota in CL and TH ducks using both the Mann-Whitney U-test (*P* < 0.01) and LEfSe analysis (LDA > 3, *P* < 0.05). Using the Mann-Whitney U-test, a detailed cecal microbiota analysis identified 16 discriminative species between the two groups strongly associated with TMA metabolism (Fig. 3A-C, Table S4). The abundance levels of *Bacteroides*, *Akkermansia*, *Bacteroides*, *Oribacterium*, *Angelakisella*, *Oscillibacter*, and *Eisenbergiella* were higher in the CL samples than in the TH samples, whereas those of *Mucispirillum*, *Sellimonas*, *Faecalitalea*, *Butyricicoccus*, *[Ruminococcus] torques group*, and *Collinsella* were lower. Additionally, LEfSe analysis revealed 25 discriminative features at the phylum (*n* = 3), class (*n* = 3), order (*n* = 3), family (*n* = 4), genus (*n* = 5), and species (*n* = 7) levels (Fig. 3D and E, Table S5). Members of the *Akkermansia* bacterial taxa were enriched in CL samples, whereas members of the *Mucispirillum*, *Olsenella*, and *Collinsella* bacterial taxa were enriched in TH samples. Both methods identified *Mucispirillum*, *Akkermansia*, and *Collinsella* as candidate bacterial taxa correlated with TMA metabolism.

In particular, *Akkermansia* and *Mucispirillum* showed the most significant differences between the CL and TH groups (Fig. 3B and D; Fig. S6A). We also observed high abundance levels of both *Akkermansia* (3.6‰, top 7.7%) and *Mucispirillum* (6.9‰, top 3.8%) (Fig. S6B). Additionally, the co-occurrence network showed that *Akkermansia* and *Mucispirillum* had limited correlation with other bacteria (Fig. S6C). Therefore, we further explored the relationships between the main candidate bacteria and other microbiota. We identified two major enterotypes *Bacteroides* and *Desulfovibrio* from the cecal samples, and revealed that the abundance levels of *Akkermansia* and *Mucispirillum*, and TMA concentration did not differ between the two enterotypes (Fig. S6D and E).

### Cecal microbes correlate with liver proteins in ducks

**Fig. 4:**
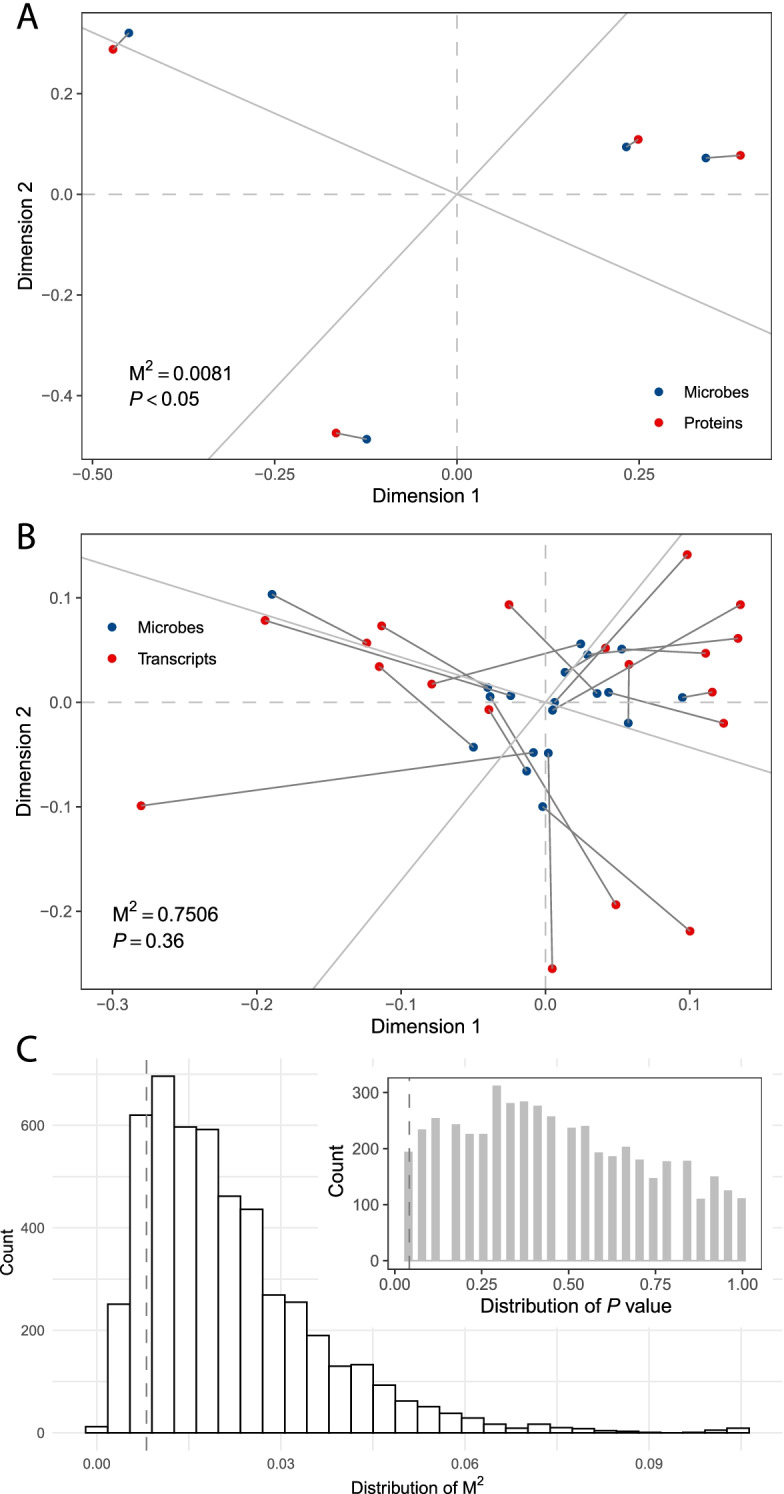
Cecal microbes had a higher correlation with proteins compared with transcripts in the liver. **A** Procrustes analysis showing the correlation between cecal microbes and liver proteins. **B** Procrustes analysis showing the correlation between cecal microbes and liver transcripts. **C** Randomly subsampled four samples to perform Procrustes analysis (microbes vs. transcripts) with 5000 permutations (without replacement) to show the validity of the Procrustes analysis between microbes and proteins. The vertical lines at the distribution plot of M^2^ and *P*-value represent 0.0081 and 0.05, respectively

Procrustes analysis was conducted to explore the relationship between the cecal microbial community and the protein/transcript profile. The results revealed a significant correlation (M^2^ = 0.0081, *P* < 0.05) between microbial community composition and protein profile (Fig. 4A). The correlation (M^2^ = 0.7506, *P* = 0.36) between microbial community composition and transcript profile was not significant (Fig. 4B). To assess whether the significant correlation was caused by sampling error, we randomly sampled the transcriptomic/microbial data without replacement and calculated the M^2^ and *P*-values 5000 times. In the Procrustes analysis, the possibility that the results of lower M^2^ and *P*-value were due to sampling errors was less than 0.1412 and 0.0386, respectively (Fig. 4C).

In duck liver, the 10 most highly expressed proteins accounted for 12.7% of the total protein quantity, while the 10 most highly expressed transcripts accounted for 33.8% of the total mRNA. However, the highly expressed proteins and mRNA did not overlap at the gene level (Fig. S7A). Moreover, comparisons of proteomic and transcriptomic changes showed that 6.9% of proteins and 4.8% of transcripts were differentially expressed. Certain genes showed significant differences at the proteomic and transcriptomic levels, and some displayed opposite changes in abundance (Fig. S7B).

### Liver FMO3 protease had insufficient content and enzyme activity in ducks

**Fig. 5:**
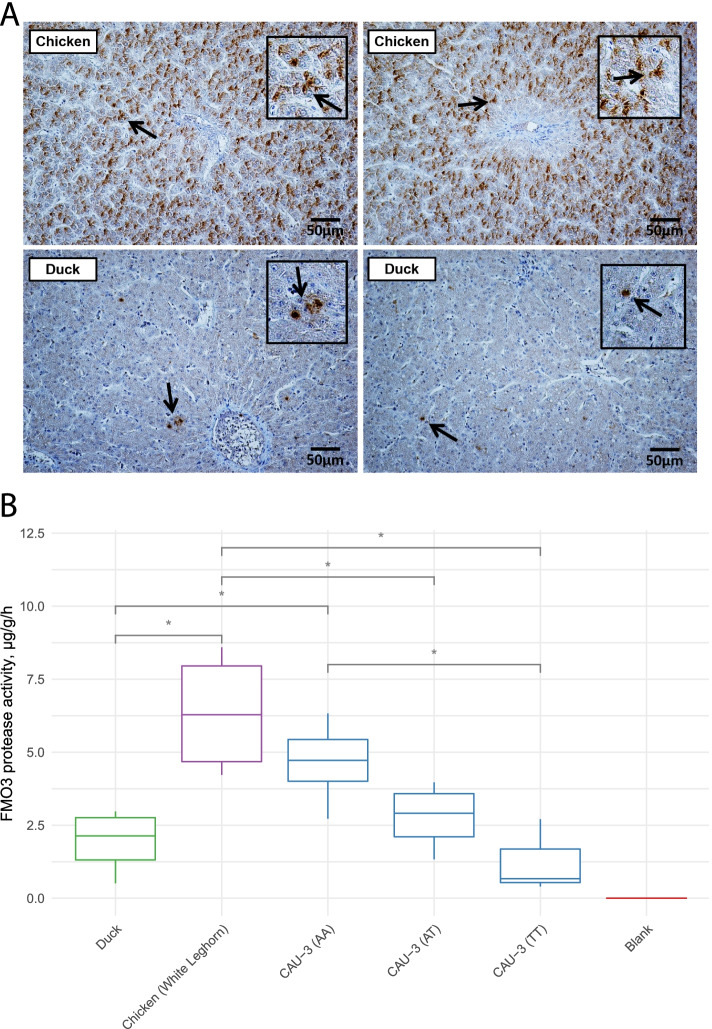
The content and activity of FMO3 protease in the liver of ducks were at lower levels compared with that of chickens. **A** Immunohistochemical (IHC) staining for FMO3 expression in chicken and duck livers, the black box represents the stained FMO3 proteins indicated by the arrows with magnification. **B** FMO3 protease activity in duck and chicken livers (Wilcoxon rank-sum test), *P < 0.05. The CAU-3 (TT) chickens have impaired FMO3 function

The immunohistochemical characteristics between chicken and duck species revealed that FMO3 protein was diffusely distributed in duck livers, while it was densely distributed in chicken livers (Fig. 5A). Additionally, the liver FMO3 enzymes activity in ducks was also lower than that in chickens (1.94 ± 1.13 μg/g/h vs. 6.35 ± 2.15 μg/g/h, *P* < 0.05), and its activity in ducks was similar to that in FMO3 functional-impaired CAU-3 (TT) chickens (Fig. 5B). These results explained the high TMA accumulation, and indicated that the liver FMO3 enzyme had limited functions regulating TMA metabolism in ducks.

### FMO3 deficiency decreased lipid deposition

**Fig. 6:**
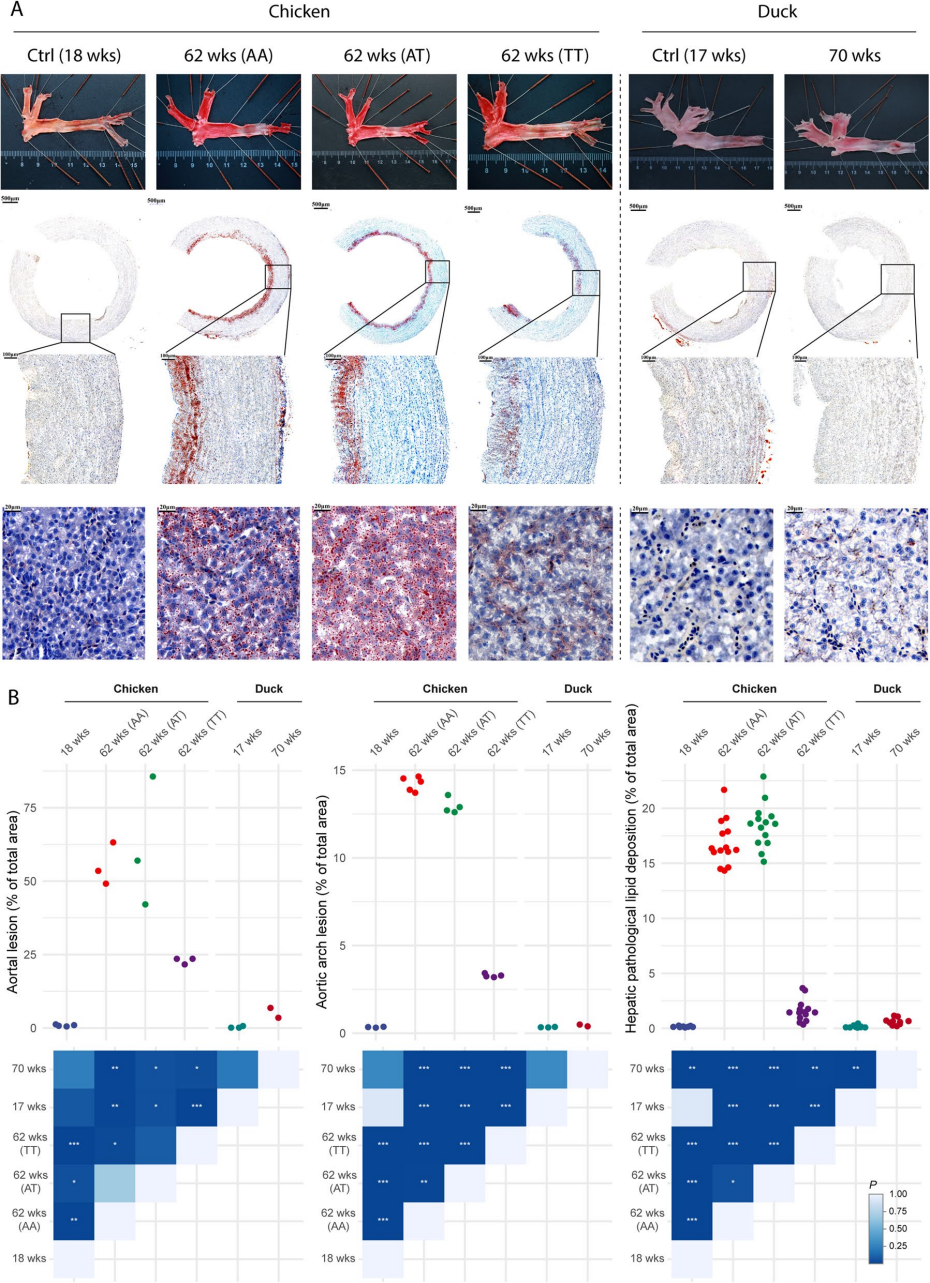
The characteristics of lipid deposition in duck’s aorta and liver were similar to that in the young and *FMO3* defected (*FMO3* c.984 A > T, TT) chicken’s aorta and liver. **A** From the top to the bottom, the three sections show Oil Red O staining of the aorta, Oil Red O and hematoxylin staining of aorta arch cross-section, and Oil Red O and hematoxylin staining of the liver, respectively. **B** From the left to the right, the three sections show quantification of staining results (% of total area) of the aorta, aorta arch, and liver (corresponding to Fig. 7A), respectively. The bottom panel shows *P*-values calculated in *t*-test, *P < 0.05, **P < 0.01, ***P < 0.001

FMO3 deficiency theoretically prohibits TMA from oxidizing to TMAO. Considering the close relationship between TMAO and CVD, we investigated the effects of FMO3 on lipid deposition. To explore the roles of FMO3 in the lipid metabolism of ducks, the histological features of atherogenesis and hepatic lipid deposition in ducks and chickens (AA, AT, and TT) were assessed (Fig. 6A and B). The results showed that lipid deposition in the aortas of both chickens (18 weeks) and ducks (17 weeks) at a young age was hardly observed. The lipid deposition indices of both aortal lesion proportion (55.3% ± 7.2% for AA, 61.6% ± 22.1% for AT) and aortic arch cross-section lesion proportion (14.2% ± 0.4% for AA, 12.9% ± 0.4% for AT) increased dramatically for chickens of genotype AA and AT (*FMO3* c.984 A > T) at 62 weeks of age. However, the aortal lesion proportion (22.9% ± 1.1%) and aortic arch lesion proportion (3.3% ± 0.1%) for chickens of genotype TT (*FMO3* impaired) were less than half of those for chickens (AA and AT). Notably, the aortal lesion proportion (5.2% ± 2.4%) and aortic arch lesion proportion (0.4% ± 0.1%) of ducks (70 weeks) were the lowest compared with those of chickens (62 weeks). Similar patterns were observed for pathological lipid deposition in the liver (Fig. 6A and B). In brief, chickens (62 weeks) of genotypes AA and AT had significantly increased lipid droplets attached to the cytoplasm of hepatocytes and evident fat droplet staining of hepatocytes in the liver. In contrast, there was almost no lipid droplet accumulation in the livers of chickens (62 weeks) (TT) and ducks (70 weeks). These results suggest that the weakened FMO3 enzyme in ducks suppressed lipid deposition in the vessel wall and hepatocytes, which had considerable advantages in lipid metabolism and would improve resistance to CVD.

### FMO3 deficiency increased antibacterial activity

**Fig. 7:**
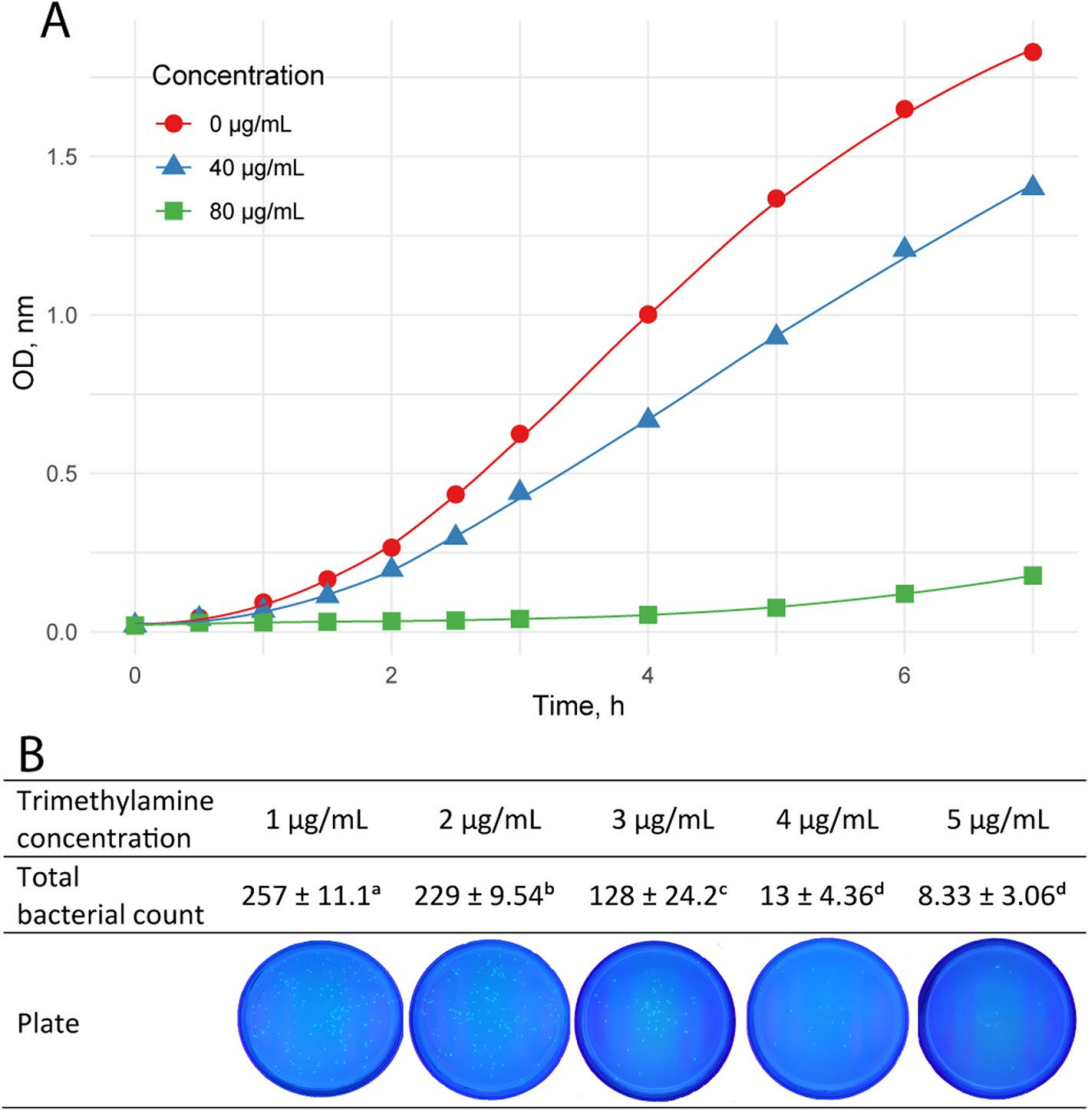
TMA inhibited the proliferation of *E. coli* in vitro. **A** The optical density (OD) of bacteria suspension with TMA concentrations of 0, 40, and 80 μg/mL, respectively. **B** Total colony counts with TMA concentrations from 1 to 5 μg/mL after 24 h. Letters (a, b, c, d) assigned after the statistic numbers represent significance. Different letters indicate a significant difference (ANOVA, Duncan test, *P* < 0.05)

Apart from the effect of FMO3 on lipid deposition, the accumulated TMA in the eggs also played a positive role in the antibacterial process. The antibacterial test showed that TMA exhibited effective antibacterial ability. Furthermore, high TMA concentrations inhibited *E. coli* proliferation (Fig. 7A). To further simulate the normal TMA concentration range in duck eggs, gradient TMA concentrations ranging from 1 to 5 μg/g were applied. The inhibition pattern was also observed; the number of colonies decreased as the TMA concentration increased (Fig. 7B).

## Discussion

To determine the unusual mechanism of TMA metabolism in ducks, this study investigated the genetic and regulatory basis of TMA metabolism in ducks and its relationship with intestinal bacteria at the gene, transcript, protein, and bacterial levels. At the transcriptional level, *FMO3* transcripts were weakly related to TMA metabolism. These results confirmed that SNPs located in the exons of *FMO3* did not correlate with TMA metabolism in ducks. Proteomic results showed that FMO3 protease regulates TMA metabolism in the liver, and may synergize with other proteins. The study found that two major intestinal bacteria (*Akkermansia* and *Mucispirillum*) regulate the preliminary TMA metabolism process at the microbial level. In addition, we found that ducks have a limited FMO3 function in regulating TMA metabolism. However, this deficiency in FMO3 improves its survival and reproductive ability.

Identifying bacteria that participate in TMA metabolism can help improve and refine the treatment options for patients with TMAU. Studies have shown that both antibiotics and probiotics can alleviate the main symptoms of TMAU by decreasing TMA levels in the gastrointestinal (GI) tract [56–59]. However, inappropriate treatment strategies may result in unpredictable side effects. For instance, antibiotics may disrupt the original intestinal flora and lead to extra diseases [60–62]. Therefore, it is essential to establish GI metabolism-targeted treatments by identifying the functions of specific bacteria. In this study, two major intestinal bacteria, *Akkermansia* and *Mucispirillum*, were negatively and positively involved in TMA metabolism, respectively. Studies have also presented similar results, indicating that *Akkermansia*, the next-generation beneficial microbe, could decrease TMA levels [63–66]. Apart from experimental evidence suggesting that *Mucispirillum* promotes TMA production, the detected putative trimethylamine N-oxide reductase (E.C. 1.7.2.3) also showed its ability to reduce TMAO to TMA [67, 68]. The high correlation between TMA and TMA metabolism-related functions indicates their potential value in treating TMAU. In the present study, similar intestinal flora communities between the case and control groups implied that only a small fraction of specific bacteria were involved in the TMA metabolism process. Their irrelevance to enterotypes further indicates that *Akkermansia* and *Mucispirillum* do not affect the overall microbial community. Moreover, these two major bacteria have limited interactions with other microbes, particularly *Akkermansia*. Therefore, treatments targeting *Akkermansia* and *Mucispirillum* would maintain the natural intestinal flora community, avoiding other side effects of intestinal flora imbalance. Probiotics such as *Akkermansia* and antibiotics targeting *Mucispirillum* are promising candidates for treating TMAU, which require further verification for their actual effects in vivo.

These results indicated that FMO3 proteases and *FMO3* transcripts participated in the downstream process of TMA metabolism, but they played limited roles in producing TMA. Both proteome and Procrustes analyses indicated that FMO3 protease directly regulates TMA metabolism accompanied by other oxidoreduction-related proteins. However, the universal fishy odor of duck eggs implies that the TMA metabolism capacity of ducks is weak, indicating that ducks might generally have mild TMAU. To further confirm this hypothesis, comparative analysis between ducks and chickens revealed that both FMO3 protease abundance and catalytic power in ducks were significantly lower than those in genetically close species. The diminished function of FMO3 is a critical factor in TMA metabolism disorders in ducks.

Further analysis revealed that the weakened metabolic capability of FMO3 reflected the evolutionary adaptability of ducks in terms of survival and reproduction. Studies in humans, livestock, and poultry have demonstrated that only a few *FMO3* gene mutations directly cause significant changes in TMA metabolism. Therefore, the possibility of weak FMO3 catalytic power occurring in humans, cows, quails, and chickens is negligible [13–15, 69]. However, ducks generally have weak FMO3 catalytic power and deficiencies in TMA metabolism. Therefore, it is reasonable to speculate that ducks undergo genetic changes to better adapt to their environment.

From the perspective of survival, ducks theoretically have a high risk of obesity and CVD, such as atherosclerosis (AS), due to their high body fat under normal conditions. However, ducks do not suffer from these health problems. In contrast, ducks are animal models resistant to AS [70]. Consistent with previous studies, our results have also shown that ducks have almost no lipid droplet accumulation in the inner and middle membranes of vessel walls compared with chickens of any *FMO3* genotype (*FMO3* c.984 A > T) [71]. AS resistance in ducks contradicts the theory that ducks have a high risk of obesity and CVD. This reflects the positive role of the weak FMO3 catalytic power in ducks. The FMO3 enzyme with physiological activity oxidizes TMA into TMAO, identified as a novel and independent marker of CVD, and explains 11% of the lesions for AS in humans and mice [72–74]. Antisense oligonucleotide (ASO)-mediated *FMO3* knockdown decreased TMAO levels and retarded AS development in mice, proving the role of this pathway in CVD [73, 75, 76]. Our study showed that AA and AT chickens with physiological FMO3 enzyme activity had a higher percentage of AS lesions. In comparison, the decreased TMA oxidation rate resulting from FMO3 protease deficiency kept ducks at a low TMAO level, thereby reducing the risk of CVD and improving their survival to some extent. Functional defects in FMO3 led to decreased lipid deposition in the vessel wall and liver, indicating that FMO3 deficiency alleviated the adverse effects of high body fat, which is beneficial to the survival of ducks.

From the perspective of reproduction, ducks instinctively lay their eggs in nests near ponds or grasses. However, various microbes, including pathogenic bacteria, tend to multiply in moist environments, posing a great threat to the incubation process [77–80]. We identified TMA in duck egg white and its egg yolk and found that TMA has an antibacterial effect in vitro. This is in accordance with earlier studies, which showed that TMA solutions reduce the survival of certain bacteria [81, 82]. TMA, derived from the weak metabolic capability of FMO3, was deposited in the egg yolk and white of ducks. It constitutes a component of the antibacterial system in eggs accompanying other well-known substances such as lysozyme and hence contributes to improving duck reproduction ability. Therefore, the weakening of FMO3 metabolism in ducks reflects their evolutionary adaptability in terms of survival and reproduction.

## Conclusions

This study investigated TMA metabolism using ducks as an animal model. In ducks, both cecal bacteria and liver FMO3 participate in TMA metabolism. Although FMO3 downregulated TMA deposition in duck liver, it had limited and less efficient functions. In the view of evolutionary adaption, the weakening of FMO3 metabolism ability improved the duck’s survival and reproduction ability in the face of ecological challenges posed by the environment.

## Supplementary Information

**Additional file 1: Fig. S1.** Data processing for weighted correlation network analysis (WGCNA) and Gene ontology enrichment categories for transcripts in the most significantly correlated module #7EC563. **A** Analysis of network topology for 1-20 soft-thresholding powers. **B** Clustering dendrogram of transcripts, with dissimilarity based on the topological overlap, and assigned merged and original module colors. **C** Gene ontology enrichment categories for transcripts in the most significantly correlated module #7EC563.

**Additional file 2: Fig. S2.** Differentially expressed transcripts were not enriched in any molecular function annotations. **A** MA plot of the quantified transcripts, and Venn diagram displaying differentially expressed transcripts. **B** Heatmap of hierarchical clustering based on the differentially expressed transcripts. **C** Gene ontology enrichment categories of the differentially expressed transcripts.

**Additional file 3: Fig. S3.** GWAS analysis based on variations at the exon level showed that SNPs located in the exon region of *FMO3* were not associated with TMA production. The top panel shows the Manhattan plot of the GWAS analysis, and the bottom panel shows the *FMO3* gene region.

**Additional file 4: Fig. S4.** TMT10-plex proteomic data processing and protein interaction networks. **A** Characteristics of the detected peptides. The left panel represents the distribution of peptide length, and the right panel represents the distribution of missed cleavages. **B** Number of peptides in each sample. **C** Normalized data distribution before and after normalization. **D** Protein interaction network of differentially expressed proteins in eight modules.

**Additional file 5: Fig. S5.** Bacteria in the duck cecum exhibited similar patterns in the CL and TH groups. **A** Rarefaction curve of the number of observed operational taxonomic units (OTUs) from 16S rRNA sequence data. **B** Histogram showing the distribution of the phyla. **C** Principal coordinates analysis (PCoA) with phylogeny-based (UniFrac) weighted distances of bacterial communities present in the duck cecum.

**Additional file 6: Fig. S6.** Characteristics of *Akkermansia* and *Mucispirillum*, and their relationships with other microbiota. **A** Spearman correlation of adjusted relative abundance for bacteria between the CL and TH groups, adjusted relative abundance = ln(abundance+Δ), Δ = 10^− 7^. **B** Cumulative abundance distribution of cecal bacteria. **C** Co-occurrence networks associated with *Akkermansia* and *Mucispirillum* (*ρ* > 0.4, *P* < 0.05). **D** Principal coordinates analysis (PCoA) using a distance matrix of enterotypes (*Bacteroides* and *Desulfovibrio*). **E** Characteristics of *Akkermansia*, *Mucispirillum*, and egg TMA contents in different enterotypes.

**Additional file 7: Fig. S7.** Only a few proteins and transcripts have potential impacts on TMA production. **A** Ranked abundance plot of proteins and transcripts in duck livers. The top 10 most abundant proteins accounted for 12.7% of the total proteins, and the top 10 most abundant transcripts covered 33.8% of all transcripts in this tissue. **B** Changes in protein and transcript abundances. The upper-right panel shows the proportion of significantly altered proteins and transcripts.

**Additional file 8: Table S1.** FMO3 mRNA expression in the CL and TH groups.

**Additional file 9: Table S2.** Differently expressed protein applying TMT10plex.

**Additional file 10: Table S3.** Gene Ontology overrepresentation enrichment of differentially expressed proteins.

**Additional file 11: Table S4.** Taxa associated with TMA metabolism using the Mann-Whitney U-test.

**Additional file 12: Table S5.** Taxa associated with TMA metabolism using LEfSe analysis.

## Data Availability

All data generated and/or analysed during the current study are available as outlined here. The raw and processed RNA-Seq data have been deposited in the NCBI Gene Expression Omnibus (GEO) under accession number GSE190019. The raw mass spectrometry proteomics data have been deposited to the ProteomeXchange Consortium via the PRIDE partner repository with the dataset identifier PXD030109. The raw 16S rRNA gene amplicon sequencing data have been deposited in the NCBI Sequence Read Archive (SRA) under accession number PRJNA785117.

## Abbreviations

TMA: Trimethylamine
TMAU: Trimethylaminuria
FMO3: Flavin-containing monooxygenase 3
TMAO: Trimethylamine N-oxide
CVD: Cardiovascular disease
HS-GC: Headspace gas chromatography
TMT: Tandem mass tags
LC-MS/MS: Liquid chromatography-mass spectrometry
*E. coli*: *Escherichia coli*
TCA: Trichloroacetic acid
IHW: Independent Hypothesis Weighting
VST: Variance stabilizing transformation
TOM: Topological overlap matrix
GI: Gastrointestinal
GS: Gene significance
MM: Module membership
TCEP: Tris (2-carboxyethyl) phosphine
IAA: Iodoacetamide
TEAB: Triethylammonium bicarbonate
RPLC: Reversed-phase liquid chromatography
HCD: Higher-energy collision dissociation
PSM: Peptide-spectrum matches
CONSTANd: Constrained Standardization
GO: Gene Ontology
PPI: Protein-protein interaction
PCoA: Principal coordinates analysis
JSD: Jensen-Shannon divergence
PAM: Partitioning Around Medoids
CH: Calinski-Harabasz
DEPs: Differently expressed proteins
DETs: Differently expressed transcripts
OD: Optical density
OCT: Optimal cutting temperature
WGCNA: Weighted correlation network analysis
AS: Atherosclerosis
ASO: Antisense oligonucleotide
