# Supplementary figures and images for: FMO3 deficiency of duck leads to decreased lipid deposition and increased antibacterial activity
Source: J Anim Sci Biotechnol. 2022 Nov 16;13:119. doi: 10.1186/s40104-022-00777-1 (PMC9667675; doi:10.1186/s40104-022-00777-1)

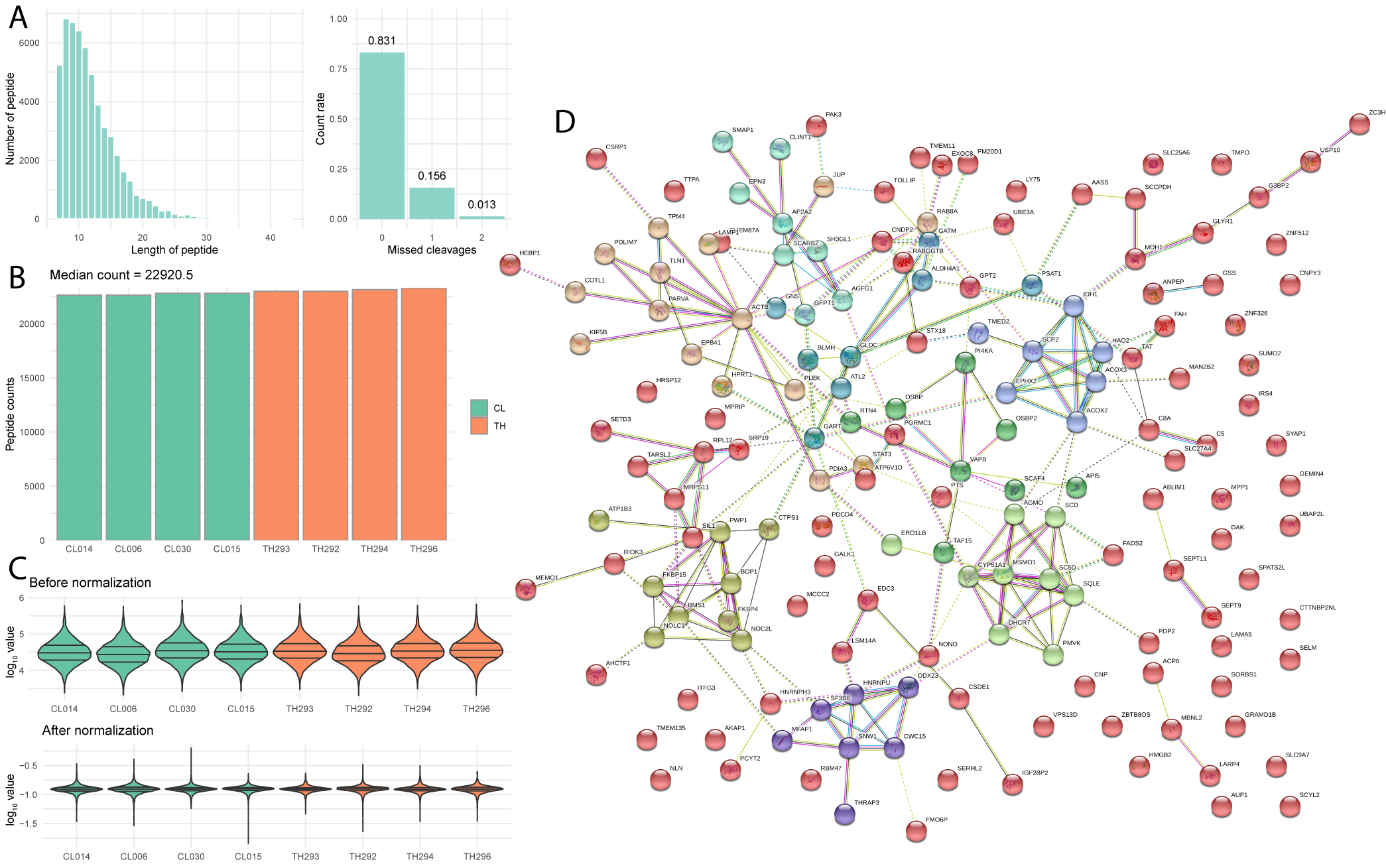

Supplement: Supplementary file 1 — Additional file 1: Fig. S1. Data processing for weighted correlation network analysis (WGCNA) and Gene ontology enrichment categories for transcripts in the most significantly correlated module #7EC563. A Analysis of network topology for 1-20 soft-thresholding powers. B Clustering dendrogram of transcripts, with dissimilarity based on the topological overlap, and assigned merged and original module colors. C Gene ontology enrichment categories for transcripts in the most significantly correlated module #7EC563. [file 40104_2022_777_MOESM1_ESM.tif]

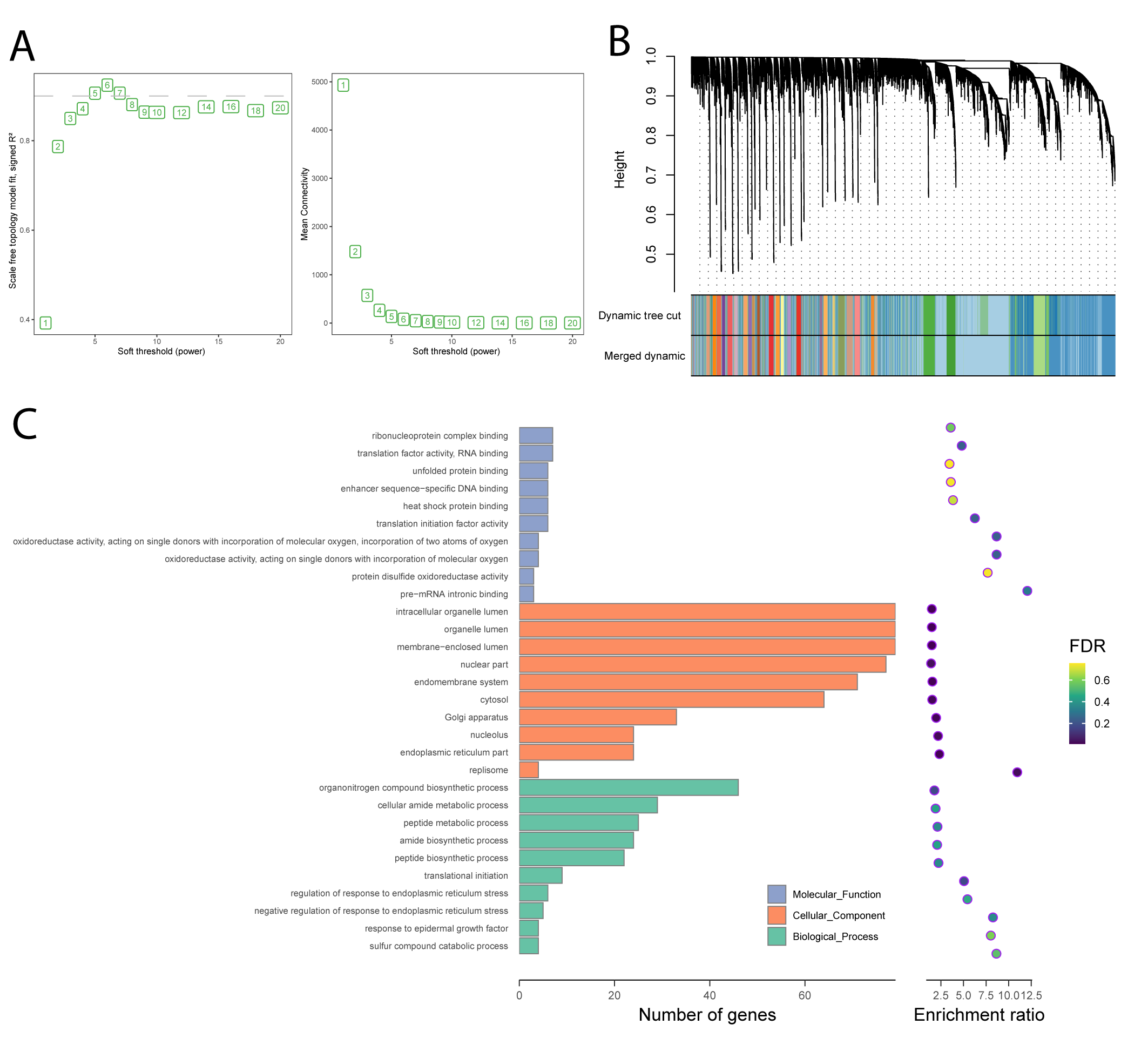

Supplement: Supplementary file 2 — Additional file 2: Fig. S2. Differentially expressed transcripts were not enriched in any molecular function annotations. A MA plot of the quantified transcripts, and Venn diagram displaying differentially expressed transcripts. B Heatmap of hierarchical clustering based on the differentially expressed transcripts. C Gene ontology enrichment categories of the differentially expressed transcripts. [file 40104_2022_777_MOESM2_ESM.tif]

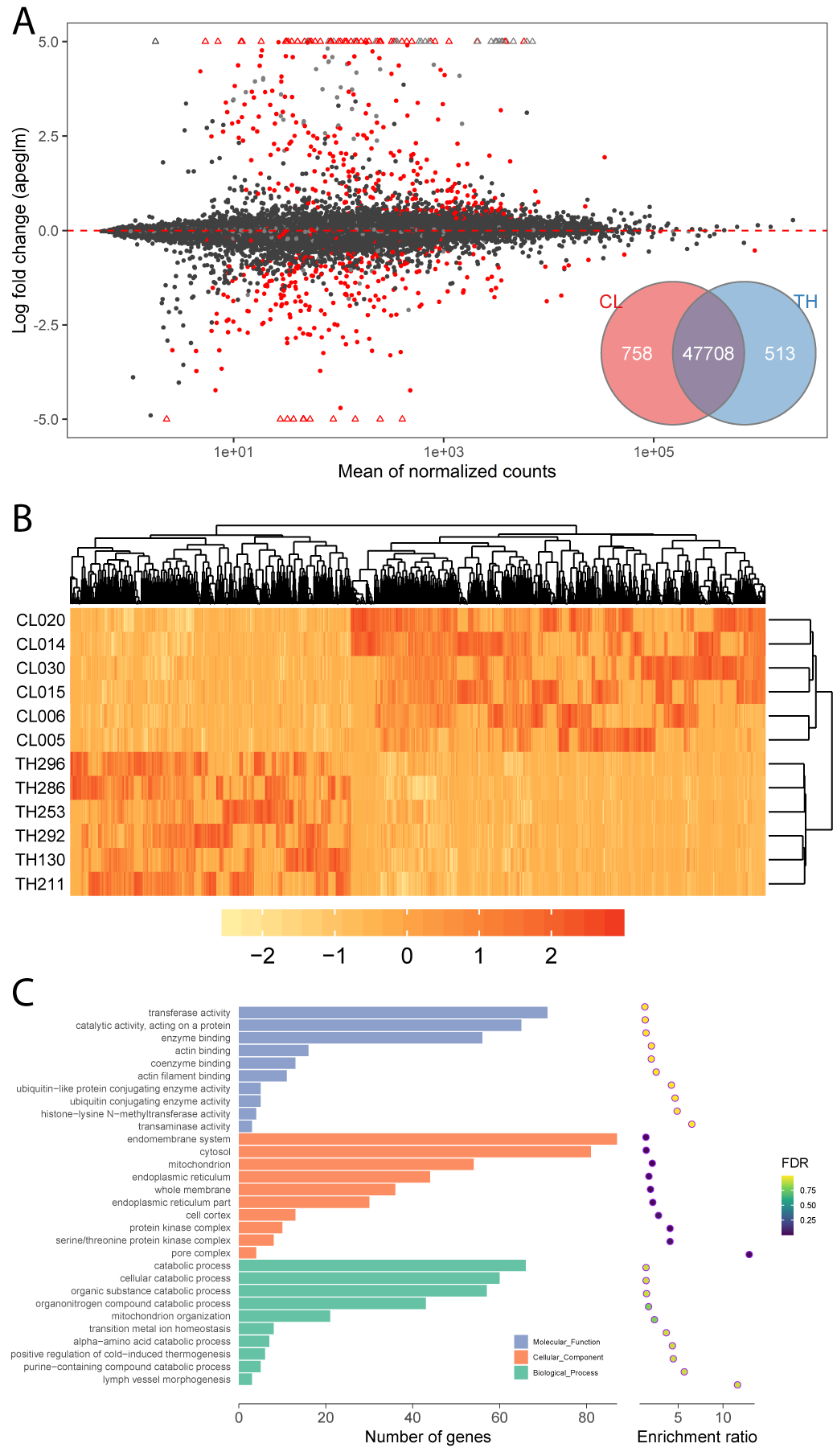

Supplement: Supplementary file 3 — Additional file 3: Fig. S3. GWAS analysis based on variations at the exon level showed that SNPs located in the exon region of FMO3 were not associated with TMA production. The top panel shows the Manhattan plot of the GWAS analysis, and the bottom panel shows the FMO3 gene region. [file 40104_2022_777_MOESM3_ESM.tif]

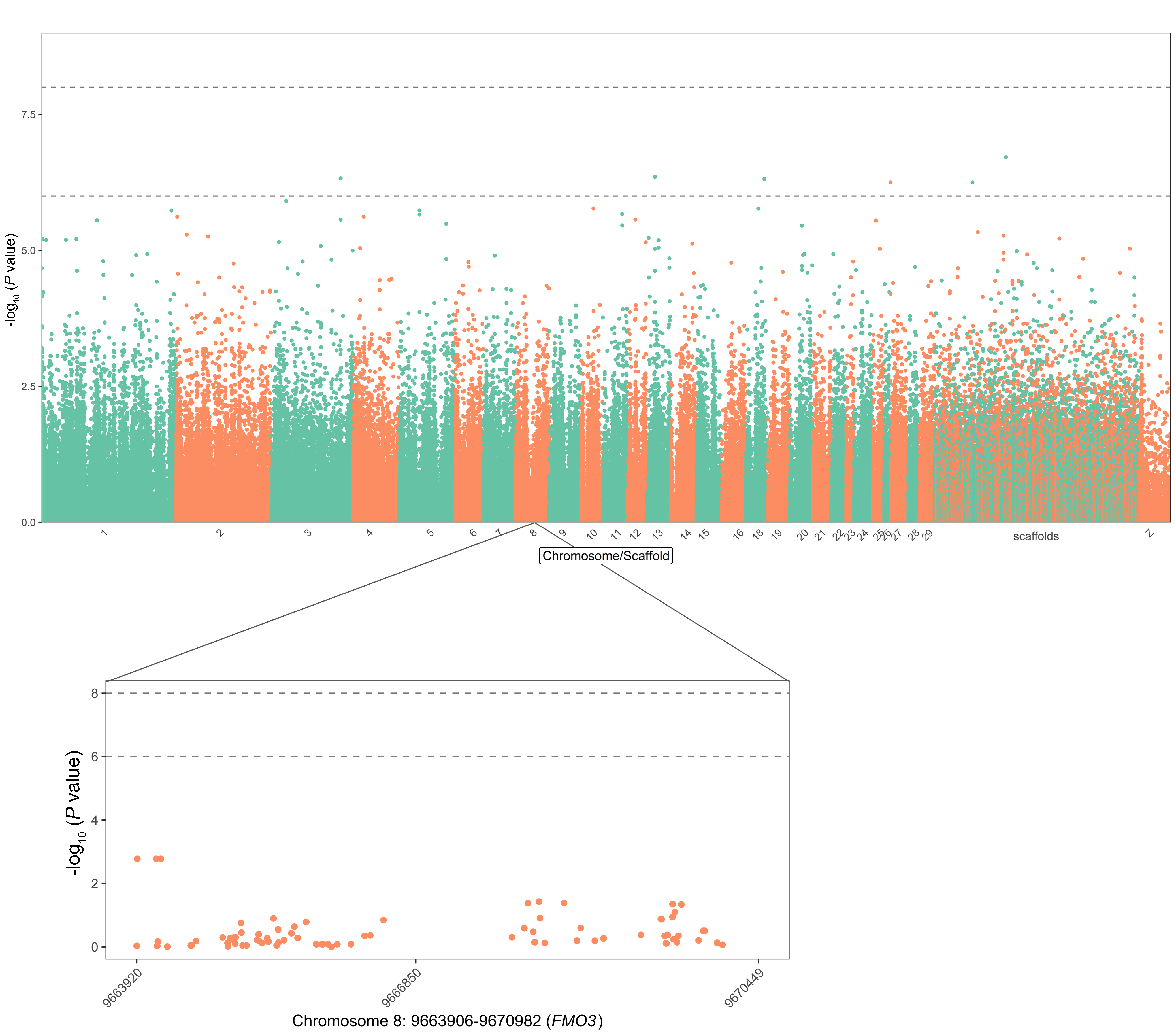

Supplement: Supplementary file 4 — Additional file 4: Fig. S4. TMT10-plex proteomic data processing and protein interaction networks. A Characteristics of the detected peptides. The left panel represents the distribution of peptide length, and the right panel represents the distribution of missed cleavages. B Number of peptides in each sample. C Normalized data distribution before and after normalization. D Protein interaction network of differentially expressed proteins in eight modules. [file 40104_2022_777_MOESM4_ESM.png]

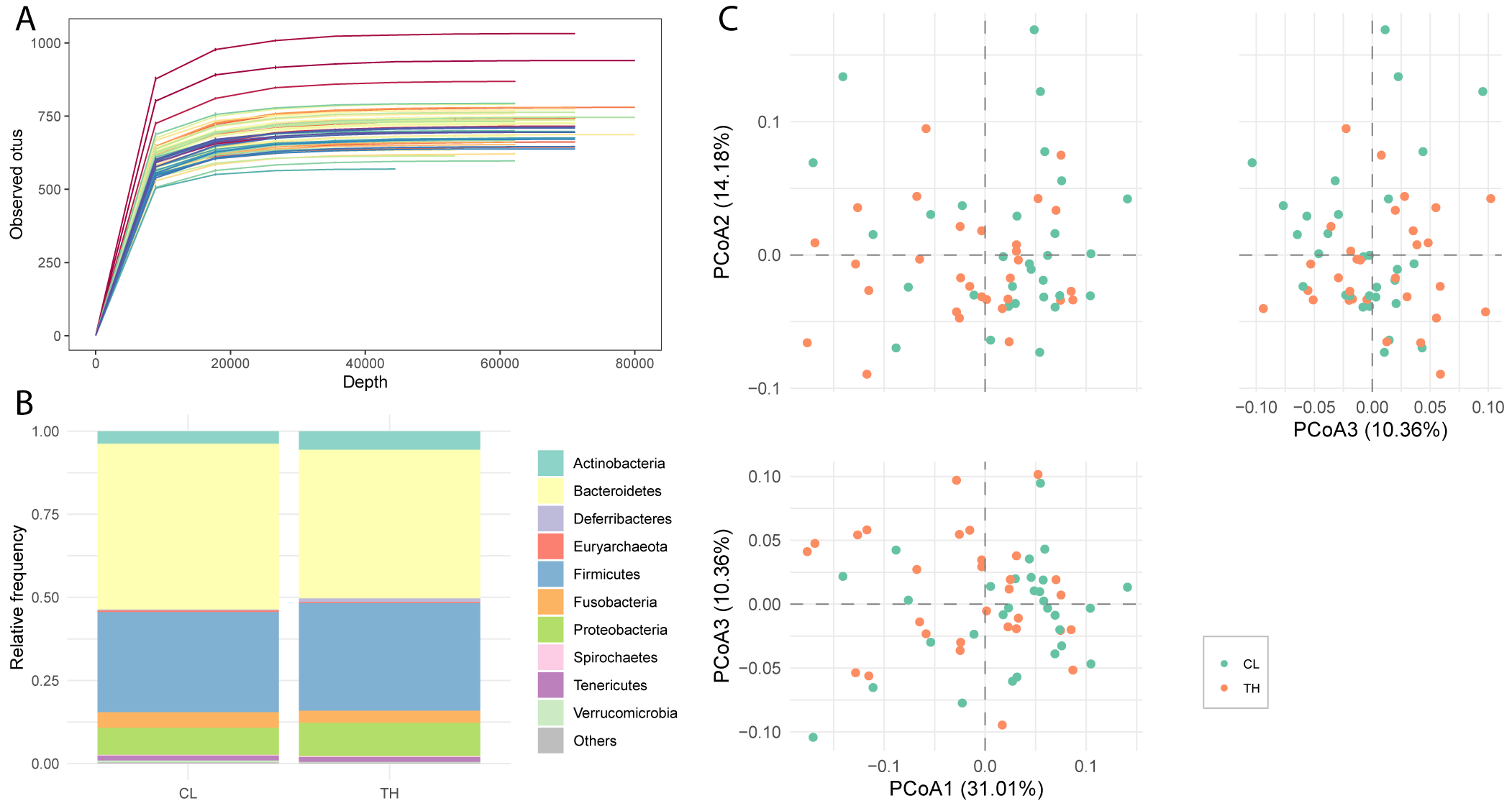

Supplement: Supplementary file 5 — Additional file 5: Fig. S5. Bacteria in the duck cecum exhibited similar patterns in the CL and TH groups. A Rarefaction curve of the number of observed operational taxonomic units (OTUs) from 16S rRNA sequence data. B Histogram showing the distribution of the phyla. C Principal coordinates analysis (PCoA) with phylogeny-based (UniFrac) weighted distances of bacterial communities present in the duck cecum. [file 40104_2022_777_MOESM5_ESM.tif]

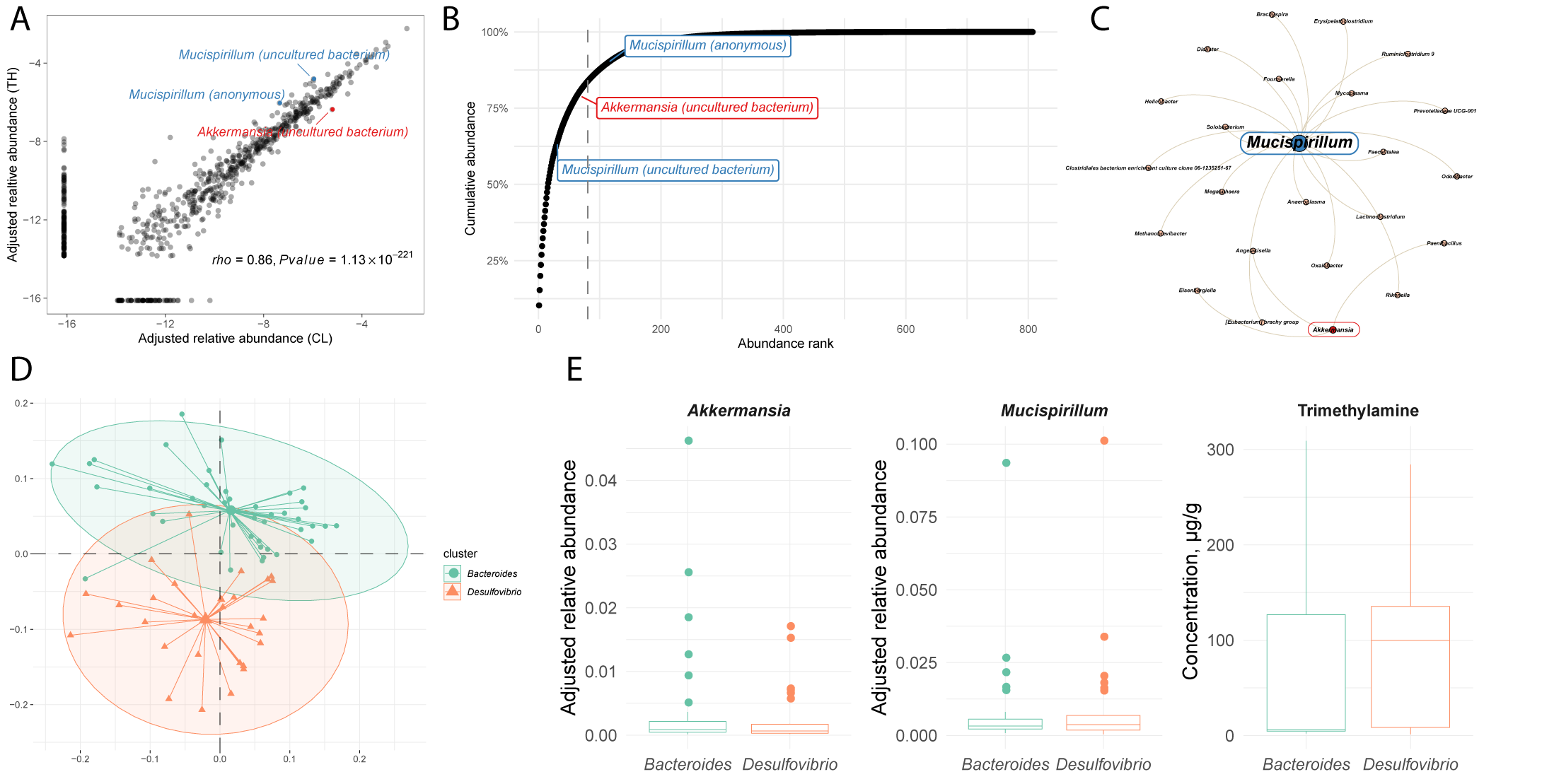

Supplement: Supplementary file 6 — Additional file 6: Fig. S6. Characteristics of Akkermansia and Mucispirillum, and their relationships with other microbiota. A Spearman correlation of adjusted relative abundance for bacteria between the CL and TH groups, adjusted relative abundance = ln(abundance+Δ), Δ = 10− 7. B Cumulative abundance distribution of cecal bacteria. C Co-occurrence networks associated with Akkermansia and Mucispirillum (ρ > 0.4, P < 0.05). D Principal coordinates analysis (PCoA) using a distance matrix of enterotypes (Bacteroides and Desulfovibrio). E Characteristics of Akkermansia, Mucispirillum, and egg TMA contents in different enterotypes. [file 40104_2022_777_MOESM6_ESM.tif]

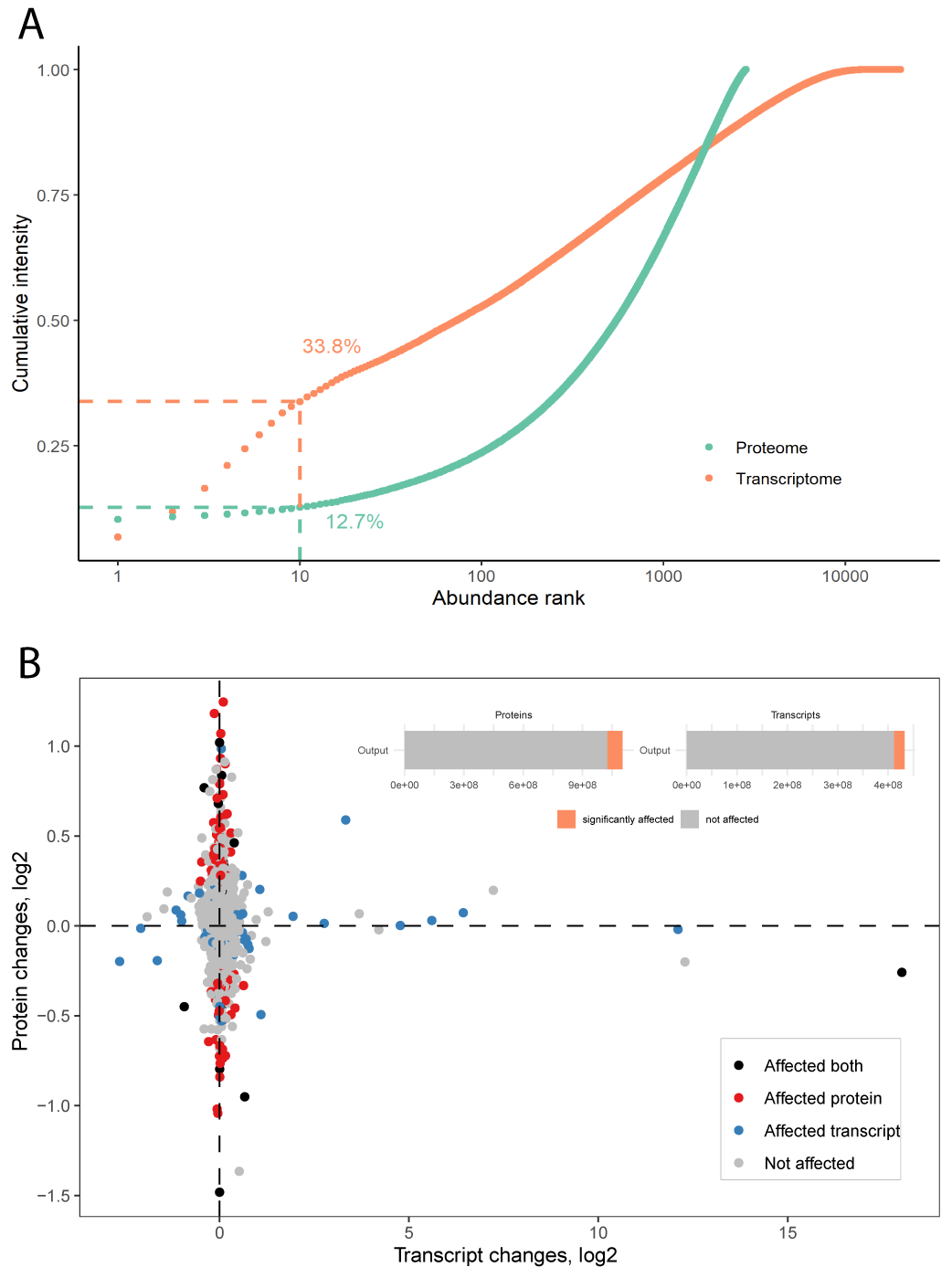

Supplement: Supplementary file 7 — Additional file 7: Fig. S7. Only a few proteins and transcripts have potential impacts on TMA production. A Ranked abundance plot of proteins and transcripts in duck livers. The top 10 most abundant proteins accounted for 12.7% of the total proteins, and the top 10 most abundant transcripts covered 33.8% of all transcripts in this tissue. B Changes in protein and transcript abundances. The upper-right panel shows the proportion of significantly altered proteins and transcripts. [file 40104_2022_777_MOESM7_ESM.tif]
